# Real-time detection of rare roadside obstacles using YOLOv8-n in autonomous vehicles

**DOI:** 10.1371/journal.pone.0350732

**Published:** 2026-06-12

**Authors:** Afeera Bint-e Tanveer, Muhammad Ayoub Kamal, Muhammad Mansoor Alam, Mazliham Mohd Su’ud

**Affiliations:** 1 Faculty of Computing and Informatics (FCI), Multimedia University, Cyberjaya, Malaysia; 2 Department of Software Engineering, National University of Technology, Islamabad, Pakistan; 3 Department of Computer Science, DHA Suffa University, Karachi, Pakistan; 4 Department of Computer Science and Software Engineering, Riphah International University, Islamabad, Pakistan; Wuhan University of Technology, CHINA

## Abstract

Rare road obstacles, including traffic cones, fallen trees, debris, barrels, and rocks, pose significant safety risks to autonomous vehicles. This paper presents a lightweight real-time detection framework using YOLOv8-n to accurately identify such obstacles on resource-constrained hardware. Multiple open source datasets containing annotated images of rare objects were combined and curated into a unified dataset. The model was refined using transfer learning, and its resilience to changing illumination and partial occlusion was enhanced by data augmentation techniques such brightness fluctuation, rotation, flipping, and geometric distortion. On a mid-range NVIDIA P100 GPU, the model maintained an inference speed of 68 frames per second while achieving a precision of 95.4%, recall of 93.9%, F1-score of 94.6%, and mean average precision (mAP@0.5) of 98.1%. These findings show that the framework is appropriate for edge-based autonomous driving systems where low latency and computational efficiency are crucial since it provides precise real-time detection without the need for expensive hardware.

## 1. Introduction

Autonomous vehicles (AVs) are in rapid development of research projects to modern and final systems that can operate in sophisticated urban settings. The most crucial part of such a technological change is perception, which can be characterized as a vehicle being able to understand its environment and make judgments that are as sound as those by a human, but accomplished in machine-accelerated timeframes [1,2]. To ensure safe navigation, a perception system is needed to handle raw sensor data in real time, identify and localise significant objects and interpret dynamic road scenes with adequate accuracy. In the last 10 years, significant discoveries in deep learning and the computer vision field have driven significant progress in object detection, thus facilitating the safe identification of vehicles, pedestrians, and traffic lights in a large range of settings [3,4]. Such developments have been driven by large amounts of annotated data as well as more complex detection systems. Yet, beneath this impressive progress lies a critical blind spot.

Road environments are unpredictable. Alongside the usual traffic elements, AVs may have to deal with entities not very often modeled in the typical training data, including fallen tree branches, rocks, scattered construction barrels, detached mirrors, stray tires, traffic cones, or road debris of different shapes and sizes. These so called rare road side obstacles do not happen with high frequency to affect the learning process of perception models; however, when they do happen, they may have serious negative consequences [5,6]. A cone lying in an unidentified construction lane can instigate evasive measures; a wrongly graded rock could result in damage to the undercarriage; an unknown debris on a highway could be the trigger of an apocalyptic chain reaction. Such uncommon and yet high consequences events serve as an excellent reminder why the need to identify such obstacles with the same scrutiny as vehicles and pedestrians [7].

This is made more difficult by the requirements of real-life driving. AVs have to identify such obstacles in different lighting, weather, and traffic conditions, in many cases, and at significant speeds [8,9]. This they have to do on edge-computing platforms with limited memory, processing capabilities and energy capacity [8]. Such constraints do not provide much space to computationally intensive pipelines of detection. The embedded automotive platform has to walk the fine line, unlike cloud-based models where resources are virtually unlimited: the platform must be fast enough to respond immediately to road hazards, but intelligent enough to pick-out objects that the model might have encountered just a few times during training. The existing object detectors are mostly trained on MS COCO or KITTI datasets which disproportionately include common types of objects [9].

Consequently, models that perform well on benchmark leader boards can fail in the face of objects that lie out of such distributions [10]. Rare obstacles can tend to be small, non-uniform, partly covered or visual ambiguity and hence would be hard to be identified against cluttered urban backgrounds. The challenges are further increased during poor weather or during night situations where sensor noise and poor visibility further distort the rare objects. Simultaneously with the challenges on the data sets, the architectures should also be updated to support the real-time detection needs. YOLO family of models has played the most important role in this evolution, starting with simple models, and moving on to the mighty YOLOv8 architecture [11].

Recent versions of YOLO use anchor-free detection heads, multiscale fusion, and sophisticated augmentation techniques and have reached a new level of detection accuracy and speed [12,13]. Nevertheless, these models in their complete forms are computationally expensive, which makes them a major challenge to use in embedded systems. It has caused the production of lightweight detection models that are specifically intended to be applied on edge devices. YOLOv4-tiny, YOLOv5-n, and YOLOv8-n have smaller numbers of parameters and can run faster and do not need high power (power) devices to run without compromising the core detection ability [10,12–14].

Such small models are especially appealing to AVs, which have to process information on a continuous basis and with strong resource limitations. In concert with architecture, post-processing strategies and enhancement in features have also developed to provide better performance on small or difficult-to-detect objects, additional possibilities to adjust lightweight detectors to the infrequent obstacle detection problem have arisen [2,15,16].

In the meantime, domain-specific fields have proven the effectiveness of object detector architecture adaptation to particular environment-specific issues. In empirical studies of UAV imagery, agricultural robots, and autonomous ground vehicles navigation, it is demonstrated that focused architectural adjustments could lead to the improvement of small or highly dense object detection in a complex environment [9,11].

These results suggest that other domain-specific adaptations might also prove useful in roadside obstacle detection on rare roads. This is especially relevant in traffic scenarios in the case of temporary obstructions like cones and barrels and are commonly seen in construction or transition roadways and require speedy and accurate reactions by autonomous vehicles. Taken together, these trends highlight the existence of an urgent need of models that combine the efficiency of lightweight architectures and specialised functions to detect rare obstacles that are of safety critical importance.

Traditional detectors only work well on common categories that are well represented and fail to work when faced with a rare object. Rare obstacles cannot be considered edge cases, in fact, they are possible precursors to major accidents. To fill this gap, the implementation of lightweight model designs, emphasis training approaches, and real-time deployment considerations into an integrated model are needed.

The present work addresses this challenge by deliberately selecting YOLOv8-n as the foundation for rare roadside obstacle detection, owing to its favorable balance between architectural efficiency and detection capability. Rather than pursuing increased model complexity, this study investigates how a lightweight detector can be systematically adapted and optimized to address rare, safety-critical obstacles under real-world deployment constraints. The goal goes beyond the incremental refinement, requiring a more fundamental rethink on how perception systems can accommodate unexpected cases; in autonomous driving, it is often safety that depends not on recognizing prototypical or interesting cases, but with identifying rare, unusual cases.

We will endeavor to fill in some of the abovementioned gaps in this study by compiling a single cohesive list of rare roadside obstacles, and by creating a lightweight and real-time detection system that is specially purpose-built with this problem in mind. Specifically, we combine a number of publicly available datasets of rare roadside objects and use consistent annotation procedures to generate more representative and challenging dataset. To extend the YOLOv8-n architecture, we present an optimised detection framework to utilise transfer learning to adapt to the rare-object domain. To enhance the strength of the model in diverse environmental settings, we use data-augmentation techniques that include luminance modulation and geometric transformations, thus making the model more favorable to the environmental changes in terms of illumination, scale, and partial occlusion. The proposed methodology is computationally efficient, making it callable on a mid-range GPUs and edge devices that can be deployed in an autonomous driving implementation routinely.

The main contributions of this work are summarized as follows:

- A unified and carefully curated dataset of rare roadside obstacles is constructed by merging multiple open-source datasets, addressing data scarcity and class imbalance in rare-object detection.
- A lightweight real-time detection framework based on YOLOv8-n is developed and systematically optimized for rare roadside obstacle detection under edge-computing constraints.
- Robustness to real-world challenges such as illumination variation, scale changes, and partial occlusion is enhanced through targeted data augmentation strategies.
- Comprehensive experimental evaluation, including comparison with a baseline YOLOv8-n configuration, demonstrates high detection accuracy and real-time performance (68 FPS), validating the suitability of the proposed framework for autonomous driving deployment.

The remainder of this paper is organized as follows: Section 2 presents related work; Section 3 outlines the methodology; Section 4 details the experimental setup; Section 5 discusses results and analysis; and Section 6 concludes the paper with potential directions for future research.

## 2. Related work

Object detection has undergone significant development in the sphere of autonomous driving, especially the development of the YOLO (You Only Look Once) line of models [17,18], which offer a favorable balance between speed and accuracy [19,20]. First versions, i.e. YOLOv3 and YOLOv4, provided solid baseline detection characteristics with respect to generic objects. Later versions, such as YOLOv5 and YOLOv7, added improvements in processing performance and scaling, thus enabling it to be deployed on a wider platform range. The latest YOLOv8 series has achieved the state-of-the-art performance on various benchmark data sets. However, the complete versions of these models are still computationally expensive which poses a challenge to implementation in real-time on embedded and edge-based devices [21].

Several studies have evaluated the YOLOv8 framework in diverse traffic and environmental conditions. Afdhal et al. [1] YOLOv8 was tested in the mixed-traffic to test generalization under changeable conditions of illumination and occlusion. The study has shown the strength of YOLOv8 when used in a more traditional environment but it was not specifically focused on detecting less common objects on the road, including cones, debris, or temporary barriers. Khalili et al. [5] iACDC and DAWN data sets were incorporated into YOLOv8 to make it more resistant to weather conditions and performance under unfavorable environmental conditions. Although the outcomes are expected to be good in such conditions of unfriendly weather, the research orientation was rather on the environmental variability with minimal emphasis being laid on the infrequent roadside objects. Liang et al. [6] evaluated an edge cloud offloading scheme of YOLO-based detection, which is a computationally efficient pipeline of road scene interpretation. However, the research focused mainly on the efficiency of architectural structures and the specialized recognition of unusual objects was given minimal attention.

Other research has investigated lightweight YOLO variants for speed–accuracy trade-offs. Terven et al. [19] The small-object and small-object detection on UAV-imaging with YOLOv7-Tiny was used to achieve a good trade-off between inference speed and accuracy. Nevertheless, the model had a higher computational burden compared to YOLOv8-n. YOLO-Lite was suggested to be deployed to the extreme low-powered devices and provided significant processing speed improvements at a reasonable cost in terms of detection accuracy, particularly on small and complicated objects [21]. Padia et al. [22] employed YOLOv4-Tiny for embedded traffic monitoring, achieving improved speed compared to YOLOv3-Tiny but reporting accuracy degradation in challenging lighting and crowded urban scenes. Similarly, LittleYOLO [23] adapted YOLOv5-Nano for industrial hazard detection, demonstrating low parameter counts and suitability for edge deployment, but with reduced mAP scores and difficulty handling occluded or rare objects.

Several approaches have extended YOLO architectures to improve specific detection challenges. For instance, the work of [24] integrated YOLOv8 with AKConv and MSDA to enhance multi-scale and small object detection. Although their method improved detection in complex road scenes, the focus was not on rare or temporary roadside objects. Lead et al. [25] proposed a scale-sequence fusion mechanism with YOLOv8 for tiny object detection, but their application domain excluded temporary roadside hazards like cones or debris. Amw et al. [26] directly addressed rare object detection by optimizing YOLOv8 for debris identification; however, their framework lacked adaptations for lightweight, real-time performance on autonomous vehicle platforms. Similarly, Adverse et al. [27] applied YOLOv8 to road hazard classes, focusing on surface-level damage detection such as potholes rather than physical obstacles. Conical et al. [28] used YOLOv8s for the detection of conical buckets and other uncommon objects, but their work did not discuss lightweight model deployment or edge inference optimization. Finally, Arnold et al. [29] tested the YOLOv8-n model both on the COCO dataset and the traffic dataset and found a significant amount of accuracy could be achieved with very low inference latency without fine-tuning to the detection of exceptional roadside obstacles. The literature shows that the series of YOLO architectures are systematically enhanced in terms of their detection accuracy, computational power and methods of feature-extraction.

Overall, the literature reveals a clear progression in YOLO architectures from v3 through v8, with each iteration offering improvements in detection accuracy, speed, and feature extraction strategies. However, most prior works either focus on improving detection accuracy for common traffic elements or optimizing computational efficiency in isolation, without specifically addressing the detection of rare roadside obstacles in real time [24,25]. Lightweight versions such as YOLOv5-s or YOLOv8-s improve deployability but often exhibit reduced robustness for small or infrequent objects. Lightweight versions such as YOLOv5-s or YOLOv8-s improve deployability but often exhibit reduced robustness for small or infrequent objects [26,30]. The specified gap inspires the current study to use YOLOv8-n, which is a smaller framework with a lower number of parameters, taking advantage of its ability to fine-tune on scarce obstacle samples. This method guarantees resource constrained systems of true and real-time detection.

Table 1 presents the summaries of the chosen works that used different variants of YOLO, identifies their methodology, the problem being solved, and limitations they encountered. This comparative study explains the existing trade-off among accuracy, computation efficiency, thus it forms the basis of the justification of using YOLOv8-n in the current study to reach the best results on resource-limited systems.

**Table 1:** Summary of YOLO-based methods for rare roadside obstacle and real-time detection.

Reference	Methodology used	Challenges addressed	Limitations
[1]	YOLOv8 evaluation across mixed traffic	Lighting and occlusion generalization	No focus on rare objects like debris/cones
[5]	YOLOv8 with merged ACDC & DAWN datasets	Weather robustness	Focus is on weather not low-frequency roadside objects
[6]	YOLO with edge-cloud offloading	Compute-efficient detection	No focus on rare roadside obstacle detection
[19]	YOLOv7-Tiny for UAV small object detection	Balanced speed and accuracy	Higher computation cost than YOLOv8-n; still heavier model size
[21]	YOLO-Lite on VOC dataset	Extremely lightweight for microcontrollers	Significant drop in detection accuracy, especially small objects
[24]	YOLOv8 + AKconv + MSDA	Small/multi-scale object detection	Focuses on road scenes, not rare/temporary objects
[25]	YOLOv8 with scale-sequence fusion	Tiny object detection	No application to cones, debris or temporary roadside objects
[26]	YOLOv8 optimized for debris	Rare object detection	Lacks lightweight implementation for AV systems
[27]	YOLOv8 on road hazard classes	Surface hazard detection	Surface-level damage only, not physical obstacles like cones
[28]	YOLOv8s for conical bucket detection	Uncommon object detection	Lacks edge/lightweight system discussion
[31]	COCO datasets	Fast detection on low-power devices	Poor small object accuracy; outdated feature extraction
[22]	YOLOv4-Tiny for embedded traffic monitoring	Speed improvement over YOLOv3-Tiny	Reduced accuracy in complex lighting and crowded scenes
[23]	YOLOv5-Nano for industrial hazard detection	Low parameter count; deployable on edge	Lower mAP than YOLOv8-n; struggles with rare/occluded objects
[29]	YOLOv8-n (Nano) for COCO & traffic datasets	High accuracy for small model; fast inference; optimized architecture	Not yet trained specifically for rare roadside objects; requires fine-tuning

Despite the substantive advancement seen throughout the YOLO family and throughout the vastly varied domain-specific adaptations, there still remains a consistent gap in the effective recognition of the low-frequency roadside hazards and, at the same time, maintaining the real-time operation on resource-limited systems. The literature then is either focusing on accuracy of conventional traffic objects, or focusing more on architectural efficiency without specific mechanisms of dealing with unusual hazards. Lightweight models are often less robust in complex and low-frequency detection applications, despite their increased deployability. This fact highlights the need to have a balanced approach combining compact architectures, focused fine-tuning, and scenario-specific optimization. In this environment, YOLOv8-n has a promising base, as it provides the necessary efficiency to be deployed to the embedded environment without losing enough representational complexity to support customizations to rare obstacle detection applications. Through fulfilling the listed literature gaps, the current work attempts to fill this gap in the literature by means of customized model adaptation and dataset optimization procedures.

## 3. Materials and methods

**Fig 1:**
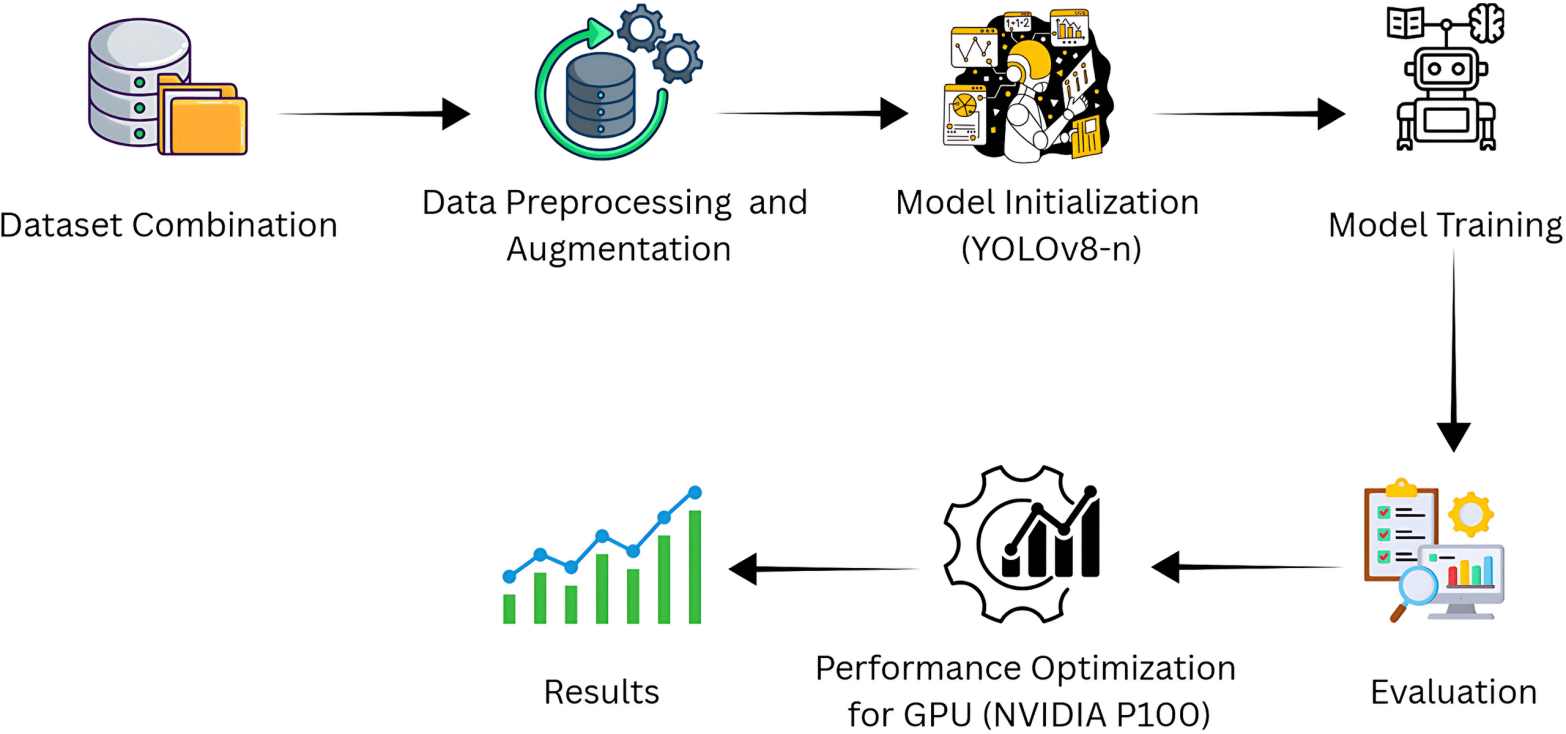
Proposed YOLOv8-n-based methodology for real-time detection of rare roadside obstacles.

The proposed system aims to achieve real-time detection of rare roadside obstacles, specifically traffic cones and debris, using a lightweight object detection model. The methodology is divided into four sequential phases, as illustrated in Fig 1.

### 3.1. Data selection and preparation

**Fig 2:**
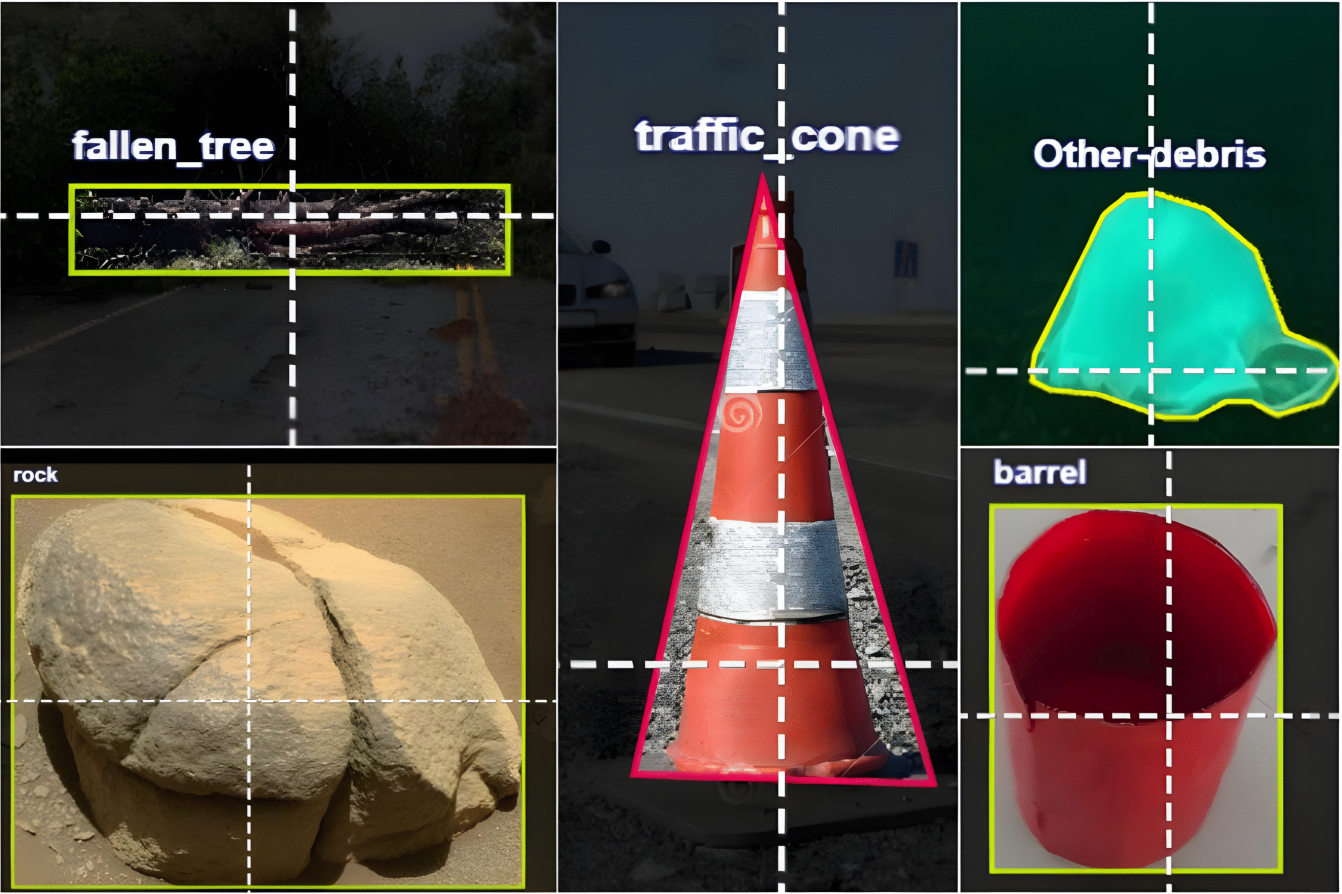
Composition of the curated dataset, integrating multiple open-source annotated datasets of rare roadside obstacles.

To construct a representative dataset for rare roadside obstacle detection, multiple publicly available datasets were integrated and curated into a unified benchmark. The dataset includes images from the following sources: (i) the Roboflow Road Debris Dataset, (ii) Traffic Cone Detection Dataset, (iii) Construction Barrel and Road Hazard Dataset, and (iv) miscellaneous roadside obstacle images collected from publicly released driving scene repositories Fig 2.

These datasets collectively cover a diverse set of environments, including urban roads, highways, construction zones, and suburban settings, captured under varying illumination and weather conditions. The images were pre-processed in order to enhance model performance and consistency. All images were first resized to a uniform resolution of 640×640. The pixel values were then normalized within the range of [0 1] as follows:


$$\begin{array}{c} I_{norm}=\frac{I-I_{min}}{I_{max}-I_{min}} \end{array}$$ (1)

where *I* represents the original pixel intensity, *I*_*min*_ and *I*_*max*_ are the minimum and maximum pixel values, and *I*_*norm*_ is the normalized pixel value. Annotation labels were carefully verified for correctness and consistency across the merged datasets. To enhance robustness against varying lighting, occlusion, and viewpoint changes, data augmentation techniques were applied, including brightness and contrast adjustment, random rotations, flipping, and geometric distortions such as scaling and shearing. Finally, the curated dataset was split into training, validation, and test subsets in the ratio 70:20:10, ensuring balanced representation for effective model training and evaluation Table 2.

**Table 2:** Composition of the curated rare roadside obstacle dataset.

Obstacle Class	Number of Images
Traffic Cones	1,850
Road Debris	1,420
Construction Barrels	1,230
Fallen Trees / Branches	980
Rocks / Obstacles	870
**Total**	**6,350**

### 3.2. Model selection and architecture

For real-time detection of rare roadside obstacles, YOLOv8-n was selected as the baseline model due to its lightweight architecture [32,33], reduced parameter count, and suitability for deployment on resource-constrained edge devices. The baseline YOLOv8-n employs a CSPDarknet-based backbone for efficient feature extraction, enabling the capture of both low-level and high-level semantic information. Let *F*_*i*_ denote the feature map extracted at stage *i* of the backbone. The extracted features can be represented as:


$$\begin{array}{c} F_{i}=\text{CSPDarknet}(I_{norm}) \end{array}$$ (2)

where *I*_*norm*_ represents the normalized input image.

The original YOLOv8-n architecture utilizes a PANet (Path Aggregation Network) neck for multi-scale feature aggregation. In this work, we further enhance the baseline YOLOv8-n by introducing a feature-weighted fusion strategy to improve the integration of multi-scale contextual information for rare obstacle detection. The proposed fused feature representation *F*_*fused*_ is formulated as:


$$\begin{array}{c} F_{fused}=\sum_{i=1}^{n}w_{i}\cdot F_{i} \end{array}$$ (3)

where *w*_*i*_ denotes the learnable fusion weight corresponding to the *i*-th feature level, and *n* represents the total number of feature scales.

The detection head of YOLOv8-n predicts bounding boxes, objectness scores, and class probabilities using an anchor-free detection mechanism, enabling accurate localization and classification of rare roadside obstacles. By incorporating the proposed feature-weighted fusion mechanism into the baseline YOLOv8-n framework, the model achieves an effective balance between detection accuracy and real-time inference speed, making it suitable for autonomous driving systems operating under limited computational resources.

### 3.3. Training procedure

YOLOv8-n was trained using transfer learning, which uses weights that are already trained on the COCO dataset to speed up training. The dataset was divided in a training set (70 percent), a validation set (20 percent) and a test set (10 percent). Rotation, horizontal flipping and switching of brightness were used as data augmentation methods to train model to be more robust to vary in light conditions, as well as partial occlusion and lead to better generalisation to real life conditions.

Training was done by minimizing the loss to correctly locate hazards (bounding boxes) and identify their type (e.g., traffic cones, debris). A 0.001 learning rate and batch size of 16 were used. The methods to avoid overfitting and to save the best-performing model were early stopping and model checkpointing. With 300 epochs the model attained high detection accuracy, but was still lightweight, and could be deployed on resource-constrained devices with high accuracy in real-time.

### 3.4. Testing and evaluation

The trained YOLOv8-n model was tested on a held-out test set of ten samples of the unified dataset. To quantitatively measure object-detection, we used standard object-detection measures: precision, recall, F1-score, and mean average precision at an intersection-over-union threshold of 0.5 (mAP@0.5). These measurements are very popular in the object-detection community and they all give a global representation of the detection accuracy and localisation capabilities of the detection system on all classes. The combination of several complementary measures provides a balanced assessment that identifies the strengths and weaknesses of the model in identifying the rare roadside impediments in different weather conditions.

Precision is the ratio of correctly detected objects to the number of detections over which the model operates hence measures the ability of the model to reduce false positives and save computational power. It is defined as:


$$\begin{array}{c} \text{Precision}=\frac{TP}{TP+FP}, \end{array}$$ (4)

where *TP* and *FP* are the numbers of true positive and false positive detections, respectively. High precision suggests that the model is unlikely to produce a false positive (warnings to drivers about safety-critical braking when not required) and low recall means many detections are missed.

Recall measures the proportion of actual objects that are correctly detected by the model, and therefore evaluates its ability to minimize missed detections:


$$\begin{array}{c} \text{Recall}=\frac{TP}{TP+FN}, \end{array}$$ (5)

where *FN* is the count of false negatives. High recall: A high value in this metric means that the model is able to detect most of the obstacles present in the environment, a necessary condition for safety-critical autonomous systems.

It is the F1-Score which combines precision and recall into a single value by calculating their harmonic mean, hence giving equal weight to them, so that the trade-off between false positives and false negatives can be balanced.


$$\begin{array}{c} F1=\frac{2\cdot \text{Precision}\cdot \text{Recall}}{\text{Precision}+\text{Recall}}. \end{array}$$ (6)

This measure is particularly beneficial in those situations when accuracy and recall are equally important, which often applies to obstacle-detection problems where false-alarms and missed detection may be equally costly.

Lastly, the mean Average Precision at a specific IoU threshold of 0.5 (mAP 0.5) is the average performance of the precision-recall curve of all classes:


$$\begin{array}{c} mAP@0.5=\frac{1}{N}\sum_{i=1}^{N}AP_{i}, \end{array}$$ (7)

where *N* is the number of object classes and *AP*_*i*_ is the Average Precision for class *i*. The mAP metric provides a holistic measure of both localization accuracy and classification performance, and is considered the primary benchmark for object detection models. By using these four complementary metrics together, the evaluation comprehensively captures the YOLOv8-n model’s ability to detect, classify, and localize obstacles under varying conditions.

### 3.5. Inference speed evaluation

Besides detection precision, model inference speed was also measured in order to establish its suitability for real-time usage in resource-constrained environments. The testing was carried out on GPU architectures using the same NVIDIA Tesla P100 accelerator used at training time. To ensure the robustness of model results, benchmarking was carried out by estimating the average time taken per image inference and corresponding number of frames per second (FPS) in multiple test iterations. This test is highly valuable for self-driving and Intelligent Road Side Monitoring systems, where it is critical for implementing timely low-latency predictions for making timely decisions.

According to the tests for inference, the YOLOv8-n model can achieve very fast and stable prediction speeds compared to other models without compromising detection. It has a very slim design that allows efficient use of its gpu resources and real-time application without very much loss of performance. Furthermore, this model is relatively small and can be deployed on edge devices like Raspberry Pi and NVIDIA Jetson Nano, which means that processing can be performed on the edge without depending on cloud services. This aspect is very much needed for autonomous or semi-autonomous vehicles that have to work under very constrained connectivity conditions, where a fast local decision is crucial.

Overall, the combined use of rigorous accuracy metrics and real-time inference evaluation provides a robust understanding of the YOLOv8-n model’s operational capabilities. High mAP and F1-score values, coupled with low-latency inference, make the model a strong candidate for practical applications in intelligent transportation systems and real-time road safety monitoring.

**Algorithm 1. Fine-Tuned YOLOv8-n Framework for Real-Time Rare Roadside Obstacle Detection**

**Require:** Curated rare roadside obstacle dataset 𝒟, COCO-pretrained YOLOv8-n model *M*_pre_, augmentation operations 𝒜, training hyperparameters Θ

**Ensure:** Trained detection model M^*^ and performance metrics {Precision,Recall,F1-score,mAP@0.5}

1: **Step 1: Dataset Preparation**

2: Collect and merge multiple open-source datasets relevant to roadside obstacles

3: Remove duplicate, corrupted, and low-quality samples

4: Resize and normalize all images to a fixed resolution

5: Apply data augmentation 𝒜 (rotation, brightness variation, scaling, geometric transforms)

6: Split dataset into training, validation, and test subsets

7: **Step 2: Model Initialization and Fine-Tuning**

8: Initialize YOLOv8-n with pretrained weights *M*_pre_

9: Adapt detection head to match the number of target obstacle classes

10: Fine-tune the model on 𝒟 using transfer learning

11: Optimize parameters using stochastic gradient descent with configured hyperparameters Θ

12: Monitor validation loss and detection metrics during training

13: **Step 3: Inference and Real-Time Detection**

14: **for** each input image or video frame *I* **do**

15:  Preprocess *I* (resize and normalize)

16:  Perform forward propagation using trained model *M* ^*^

17:  Generate bounding box predictions and confidence scores

18:  Apply Non-Maximum Suppression (NMS) to remove redundant detections

19:  Output final detected objects with class labels and bounding boxes

20: **end for**

21: **Step 4: Model Evaluation**

22: Evaluate detection performance on the test set

23: Compute Precision, Recall, F1-score, and mAP@0.5

24: Analyze class-wise performance using confusion matrix

25: Measure inference speed (FPS) for real-time applicability

26: **Step 5: Output**

27: Return trained model *M* ^*^ and evaluation results

The Algorithm 1 outlines the suggested concept of pipeline to be used in detecting rare road obstacles in real-time using the YOLOv8-n model. The process starts with dataset preparation, where several datasets are taken, merged, refined, and then data augmented through techniques to make the model robust. In the model-training stage, a YOLOv8-n model is trained and limited to the data being trained on, hyper-parameter optimization further ensures improved convergence and performance. To detect in real-time, each incoming video frame is pre-processed and sent to the trained model to make predictions. After that, an extra bounding-box filtering of overlapping boxes based on the non-maximum suppression is performed to keep the most confident detections. Thus, the ultimately identified obstacles are listed in an output, thus allowing the system to name and track objects like traffic cones and debris under changing environmental conditions.

## 4. Experimental setup

The proposed experimental research was thoroughly planned to help examine the effectiveness of the suggested YOLOv8-n based detection model on the Objects on Road dataset it was curated. The test environment included the hardware, software and training setup that was necessary to have a fair and repeatable determination of both detection accuracy and inference efficiency. This section provides transparency by definition of the computed environment, software stack and hyperparameter decisions, thus making it reproducible by other researchers.

### 4.1. Hardware environment

All experiments were performed on a dedicated supercomputing installation with the NVIDIA Tesla P100 GPU provides 16 GB of high-bandwidth memory and excellent parallel machine computation capabilities. Sufficient computational power was thus available for the intensive training and on-time inference tests, without being bottlenecked by the setup. The GPU was in synergy with a multi-core CPU working and 64 GB of system RAM that allowed the smooth loading of data, augmentation, and transfers between the CPU and GPU. However, this also increased the variability of resource constraints, thus giving our results a fair evaluation platform to accurately capture the capabilities of the detection framework we are proposing.

### 4.2. Software environment

The detection system was set up using the Ultralytics YOLOv8 library (version 8.3.176), which is a highly optimized development of the YOLO (You Only Look Once) design, with optimized training workflows and extensible model customization capabilities. The codebase was written in Python 3.11, thus also ensuring that it will run with modern deep learning libraries and programming language libraries. PyTorch acted as the backend for deep learning, and it provided automatic gradient computation, GPU support, and streamlined tensor operations. In addition to support for data preprocessing, model validation, and result inspection, other libraries were also used, such as OpenCV for image manipulation, NumPy for mathematical operations, and Matplotlib for visualization. The experiments ran in an environment that was fully controlled on Linux, thus also ensuring stable libraries and reproducible execution.

### 4.3. Training parameters

YOLOv8-n was trained using 300 epochs with a batch size of 16, the steady-state gradient updates and an appropriate amount of utilisation of the available GPU. The resolution of the input image was determined to 640 x 640 pixels to follow the standard YOLO setup to maintain the trade-off between the resolution of the detection and the execution time. The initial learning rate was 0.001 and then optimised with the stochastic gradient descent (SGD) with momentum, which is a well-known technique that promotes strong convergence in object classification. In the training the use of data-augmentation methods, random horizontal flipping, scaling and mosaic augmentation, was used to improve the generalisation ability of the model and its resistance to changes in object size, lighting and perspective. To prevent overfitting due to validation performance, an early-stopping criterion was used, and model checkpointing was used to keep the model with the best performance, which was selected by validation mAP. This systematic training program allowed the model to learn steadily in a discriminatory manner, stabilise convergence, and avoid unwarranted over-training.

## 5. Results and discussion

In this section, a stepwise quantitative and qualitative study of the suggested YOLOv8-n architecture of rare roadside obstacle detection is provided. The assessment is not just based on the typical performance measures, but also on model efficiency, training stability and class-wise detection behaviour, which are the main requirements to be deployed in autonomous driving systems under real-time and resource-limited conditions.

### 5.1. Overall detection performance

**Fig 3:**
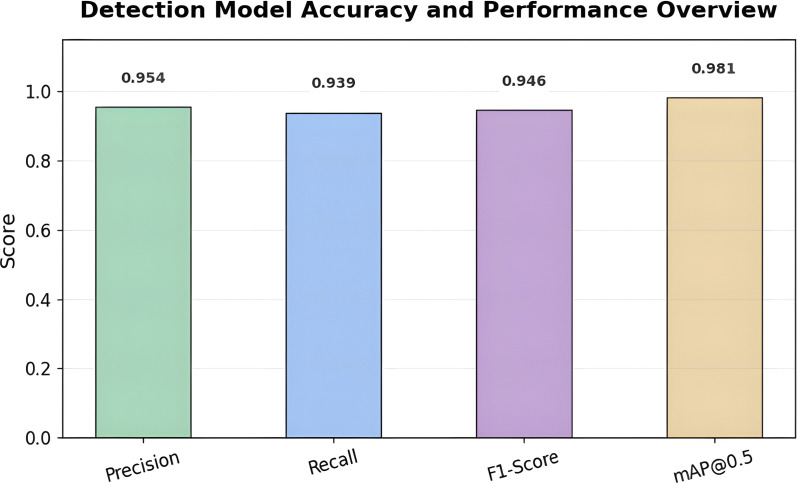
Bar graph showing YOLOv8-n detection performance across precision, recall, F1-score, and mAP@0.5.

The proposed YOLOv8-n model was tested on the curated rare roadside obstacle dataset on standard object detection measures, such as mean Average Precision (mAPO.5), Precision, Recall and F1-score, with Intersection-over-Union (IoU) of 0.5 as displayed in Fig 3

All these measures are used to measure the localisation accuracy of the model, the classification reliability, and the false positive and false negative balance.

Results show that the model has a mean average precision (mAP) of 98.1 percent at an IoU threshold of 0.5. The accuracy, the recall, and the F1-score are 95.4, 93.9 and 94.6 respectively. The fact that these are high values in all the four metrics is a pointer to a model that can efficiently detect rare roadside obstacles but at a low false-alarm rate. This is especially vital when it comes to autonomous driving, where false positives will likely result in unwarranted braking or evasive actions, but failure to detect may be life threatening.

The high recall is an indication that the model is able to identify the large majority of true obstacles situations, even those that are visually challenging and rare such as debris, traffic cones, and fallen branches. The high accuracy at the same time confirms the reliability of the detections and a lack of spuriousness of the detections. Therefore, as in F1- score, which combines accuracy and recall, provides one efficient measure of overall detection performance.

### 5.2. Real-time performance and computational efficiency

In addition to detection accuracy, real-time performance is a determining factor on useful deployment. The designed model achieves an inference rate of 68 frames per second (FPS) with NVIDIA P100 graphics card, which meets real time performance of autonomous vehicle perception systems. More importantly, the performance is achieved without adding an extra parameter to the canonical YOLOv8 architecture, which highlights the efficiency of the suggested architecture in terms of computational efficiency.

All these results verify that lightweight architectures, carefully fine-tuned and then trained on domain-specific data can at the same time meet accuracy and latency goals. Therefore, the offered framework is not only applicable to the high-performance GPUs but also to the embedded and edge-based platforms that are ubiquitous to the autonomous driving and sophisticated driver-assistance systems.

### 5.3 Baseline Comparison with Lightweight Detectors

To further support the effectiveness of the given framework, a comparative evaluation between it and the chosen lightweight and mainstream object detection models was conducted, specifically, YOLOv5s, YOLOv8s, and the vanilla YOLOv8-n. These models have been chosen due to the fact that they are popular real-time detectors that share similar design goals, model sizes, and deployment constraints. Other YOLO-based methods that were reported in the literature were tested on other datasets or tasks and thus were not included in this quantitative analysis in order to maintain fairness of the experiment.

As shown in Table 3 the proposed YOLOv8-n performs significantly better than all the baseline models using all measures of evaluation and maintains the least parameter overhead. Compared to the vanilla YOLOv8-n, the optimized model shows a significant increase in mAP 0.5, precision, recall, and F1 -score, but there is no reduction in inference speed. These findings suggest that the enhanced performance does not come as a result of more model complexity but rather of a proper curation of the datasets, transfer learning and optimization of training-strategies.

**Table 3:** Comparison of lightweight object detection models for rare roadside obstacle detection.

Model	Params (M)	FPS	Precision (%)	Recall (%)	F1-score (%)	mAP@0.5 (%)
YOLOv5s	7.2	55	89.3	87.1	88.2	90.5
YOLOv8s	11.0	60	91.7	90.2	90.9	92.4
YOLOv8-n (vanilla)	3.2	65	93.2	91.5	92.3	95.0
**Proposed YOLOv8-n**	**3.2**	**68**	**95.4**	**93.9**	**94.6**	**98.1**

Comparing to larger lightweight models like YOLOv8s and YOLOv5s, the given framework provides a better level of accuracy with a higher frame rate, thus supporting its nature as an appropriate choice in time-critical tasks. The results thus highlight the fact that the adaptation of a small detector to the rare-obstacle space can have more advantages as compared to using bigger generic networks trained on more common object categories.

### 5.4. Impact of transfer learning and data augmentation

To explain the role of certain design decisions, further experiments were performed to understand the effects of the transfer learning and data augmentation on the detection performance. Transfer learning models showed significantly reduced detection accuracy especially of small and irregularly shaped obstacles, as compared to models trained without transfer learning. This finding indicates the need to utilize pre-trained feature representations to counteract the small samples of rare-objects.

Equally, removal of data augmentation provoked decreased resistance to different illumination, change of scale and partial masking. The effect of augmentation, e.g., the change of brightness, the geometrical distortion, the flipping of the horizontal, was found to be critical in enhancing the generalization, and facilitating the model to adapt well to the real-world driving conditions. These findings affirm that both data transfer learning and goal-oriented data augmentation are part and parcel of the accomplishment of solid performance on infrequent roadside challenges.

### 5.5. Class-wise performance analysis

**Fig 4:**
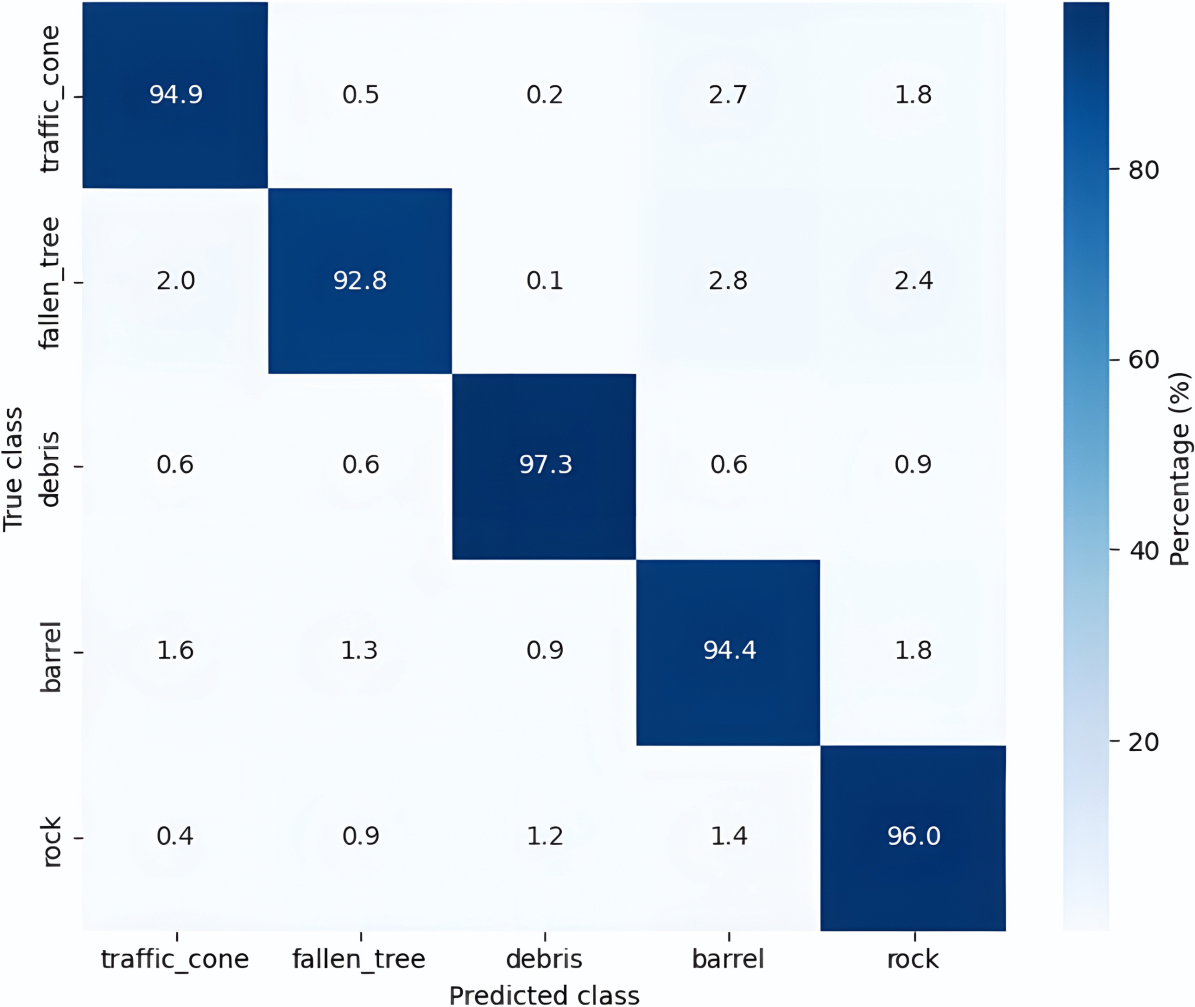
Confusion metric.

Even though aggregate metrics are useful in creating an overview, they can conceal behaviors specific to classes. To solve this, a confusion matrix was prepared to explore performance in detection in each category of obstacles. Fig 4.

The matrix is highly diagonal with majority of the classes well classified with a range of 90 98. This means that the model has acquired feature representations that are different among the types of obstacles that though they may be similar to each other visually, they are not observed very often.

Some confusion was noted between some classes like the ones of a leg and a barrel which may have similar textures and shapes in harsh light situations. This confusion is normal in real world set ups and does not play a big role in system reliability. Imperatively, the fact that the off-diagonal error is minimal, also indicates that the model gains beyond the metrics of global accuracy, and is applied to be good in detection across classes.

### 5.6. Training stability and convergence behavior

**Fig 5:**
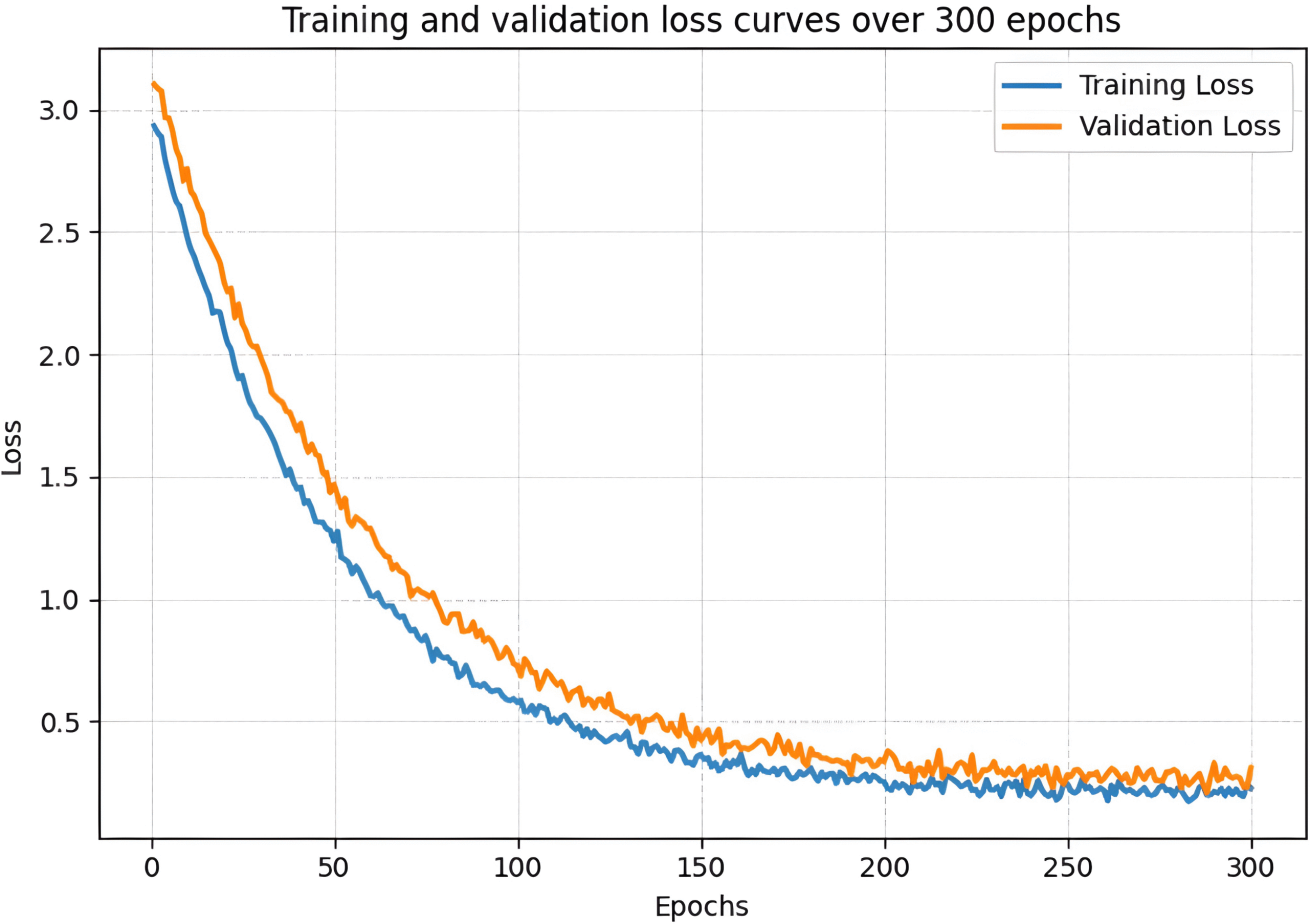
Training and validation loss curve over 300 epochs.

The proposed framework was trained with loss curves and metric curves over 300 epochs. The findings demonstrate a monotonic and smooth decline in training loss with slow increases in precision, recall, F1-score, and mAP@0.5 Fig 5. The later epochs stabilize around metric curves, which implies that the model does not overfit and is also stable.

This steady convergence behaviour implies that the model has learnt meaningful spatial and contextual characteristics of meaningful roadside obstacles, and not noise or dataset specific artifacts. This stability is necessary to be used in practice, since it means that application of the model in real-life situations will produce consistent results, even in situations where the model has not been tested in the unseen environment, or the situations of new obstacles have been observed.

### 5.7. Discussion and practical implications

On the whole, the experimental findings support the idea that the suggested YOLOv8-n framework can effectively deal with the problem of detection of the infrequent obstacles on the roadways in the real-time and under-resource conditions. The high detection accuracy, low latency, constant training behaviour, and high class-wise performance demonstrate the feasibility of the method. Compared with the vanilla YOLOv8-n architecture, the proposed model introduces a feature-weighted fusion mechanism for enhanced multi-scale feature integration. While the original YOLOv8-n employs standard PANet-based feature aggregation, the proposed approach assigns learnable weights to different feature levels, enabling the network to emphasize more informative spatial features during obstacle detection. This modification improves the representation of rare and small-scale roadside obstacles while maintaining the lightweight and real-time characteristics of the baseline YOLOv8-n framework. In a bigger context, the results indicate that the safety of autonomous-vehicles does not always need even more sophisticated models. Rather, domain sensitive dataset design, training methods, and effective model selection can be translated into significant improvements especially in low-frequency but high stakes conditions. The suggested framework signifies the direction to more than the optimized perception systems, which are also robust to the rare and unpredictable risks in the real world driving.

Despite the promising results, several limitations still remain in the current study. The proposed model was evaluated on a limited dataset and may require further validation under more diverse real-world driving conditions, including severe weather, nighttime environments, heavy traffic, and complex urban scenarios. In addition, the computational performance on different embedded and edge devices was not extensively analyzed. Future work will focus on expanding the dataset diversity, improving model generalization, and optimizing the framework for deployment in real-time autonomous driving systems with limited hardware resources. Furthermore, advanced attention mechanisms and multi-scale feature fusion strategies can be explored to further enhance the detection of rare and small-scale road obstacles.

## 6. Conclusion

This paper aims at introducing a lightweight, real-time detection system based on YOLOv8-n, which is capable of detecting any type of infrequent roadside obstacles that form a critical safety risk to autonomous cars. The framework takes advantage of a variety of open-source datasets, transfer learning, and a large amount of data augmentation methods to achieve a large detection accuracy with a low latency. Experimental evaluation provided a 95.4 percent precision, 93.9 percent recall, 94.6 percent F1 and an average mean precision at 0.5 (mAP 0.5) of 98.1 percent, with an inference rate of 68 frames per second on a mid-range NVIDIA P100 chip. The obtained results also verify the claim that the proposed model has been trained for applications on resource-constrained, edge-based self-driving systems. The subsequent studies will focus on incorporating temporal information from video streams and on expanding the dataset for representing additional sparse classes of obstacles, enhancing robustness for a vast set of real-world driving scenarios.
