# Supplementary material for: Real-time detection of rare roadside obstacles using YOLOv8-n in autonomous vehicles
Source: PLoS One. 2026 Jun 12;21(6):e0350732. doi: 10.1371/journal.pone.0350732 (PMC13262933; doi:10.1371/journal.pone.0350732)
Supplement: Attachment — Submitted filename: Rebuttal letter Plosone.docx [file pone.0350732.s002.docx]

We would like to thank the Academic Editor and the reviewers for their careful review of our manuscript and for their constructive and insightful comments. We have thoroughly revised the manuscript to address all the concerns raised. The revisions include clarification of the study’s contributions, expansion of the dataset and methodology descriptions, additional experimental analyses, and improvements in figures, tables, and formatting. All changes have been highlighted in the revised manuscript with track changes (Revised version uploaded on the portal and the screenshots just for reference).

| **Reviewer #1 Comment** | **Author Response** |
| --- | --- |
| (1) Novelty and Academic Contribution  In terms of technical originality, the manuscript primarily reflects engineering optimization and integration rather than algorithmic innovation. The authors are encouraged to clarify the originality of their contributions, for example, whether any modifications have been made to feature fusion, detection heads, loss functions, or network pruning strategies within the YOLOv8-n framework. If the approach mainly relies on fine-tuning and transfer learning, the paper could include a brief comparison table or quantitative discussion showing differences in model parameters, inference speed, and accuracy between the proposed framework and the vanilla YOLOv8-n. Additionally, the rationale for choosing YOLOv8-n instead of other lightweight versions such as YOLOv5s, YOLOv7-tiny, or MobileNet-SSD should be better articulated to strengthen methodological justification. | Thank you for this valuable suggestion. The revised manuscript now clearly highlights the originality of the proposed framework by emphasizing its task-specific optimization of YOLOv8-n for rare roadside obstacle detection under real-time and resource-constrained conditions. A quantitative baseline comparison with vanilla YOLOv8-n and other lightweight detectors has been added to demonstrate improvements in accuracy, efficiency, and inference speed. In addition, the rationale for selecting YOLOv8-n is explicitly discussed, highlighting its mature architecture and favorable accuracy–latency trade-off for autonomous driving applications. |
| (2) Dataset and Experimental Design  The dataset construction and experimental setup demonstrate a solid engineering workflow. The authors combined several open-source datasets, applied consistent image normalization and resizing, and performed data augmentation (e.g., brightness variation, rotation, and geometric transformation). However, the dataset description is somewhat vague, only stating that the images were collected from Roboflow. It is strongly recommended that the authors specify the dataset names, number of images, class distribution, and data-cleaning procedures to enhance reproducibility. If specific augmentation techniques were used, they should be explicitly described. In cases where the full dataset cannot be released, including sample statistics or representative images in an appendix would significantly improve transparency and replicability. | Thank you for this helpful comment. The manuscript has been revised to provide a detailed description of the dataset construction, including the names of the source datasets, total number of images, class-wise distribution, and data-cleaning procedures. The data augmentation strategies are now explicitly documented, and representative dataset statistics and sample visualizations have been added to improve transparency and reproducibility. |
| (3) Methodology and Algorithm Details  The methodology section is clearly written and logically organized. The description of the CSPDarkNet backbone, PANet feature fusion, and detection heads is complete, and the mathematical formulations are concise and correct. Nevertheless, Algorithm 1 appears somewhat redundant. The initialization and fine-tuning phases could be merged, and the multi-step post-processing in the inference stage could be condensed into a unified detection loop. Simplifying the pseudocode would make it more professional and readable. Moreover, adding a flowchart that visualizes the entire process, from input images to final detection outputs, would improve the paper’s technical clarity. For the transfer learning process, more training details should be provided, such as whether backbone layers were frozen and whether differential learning rates were applied during fine-tuning. | Thank you for the insightful suggestions. Algorithm 1 has been refined by merging the initialization and fine-tuning stages and simplifying the inference process into a unified detection loop for improved clarity. A flowchart illustrating the complete pipeline from input images to final detections has been added, and additional training details regarding transfer learning are now clearly specified in the methodology section. |
| (4) Experiments and Result Analysis  The experimental section is one of the highlights of this paper. The performance evaluation is comprehensive, including Precision, Recall, F1-score, and mAP@0.5, with clear visualizations of training curves and confusion matrices. The model achieved excellent results (Precision = 95.4%, Recall = 93.9%, F1 = 94.6%, mAP@0.5 = 98.1%) and demonstrated real-time inference capability at 68 FPS on an NVIDIA P100 GPU. These results strongly support the effectiveness of the proposed framework. To further strengthen the empirical analysis, the authors should include a lightweight baseline (such as YOLOv8s or YOLOv5s) for direct comparison, even if only briefly summarized. This will make the performance gain more explicit. If multiple training runs were conducted, the authors could report the mean and standard deviation; otherwise, note that the results are based on a single run. Additionally, a short ablation analysis—such as showing performance drops without data augmentation or transfer learning—would effectively demonstrate the contribution of each module. | Thank you for this encouraging feedback. The revised manuscript now includes a direct comparison with lightweight baseline models to clearly highlight performance gains in accuracy and inference speed. The experimental section also clarifies the evaluation protocol, indicating that results are reported from a single training run, and incorporates a brief ablation analysis to illustrate the individual contributions of transfer learning and data augmentation. |
| (5) Discussion  The discussion section is complete but somewhat descriptive. It would benefit from deeper analysis and interpretive commentary. The authors should not only restate the results but also explain the underlying causes of performance differences. For instance, they could discuss which design choices (augmentation strategy, fine-tuning approach, feature integration) most influenced accuracy and why some object classes (e.g., debris vs. barrels) performed differently. Including one or two examples of misdetections or false negatives, along with brief visual explanations (e.g., due to occlusion or poor lighting), would substantially enhance the paper’s credibility. The future work paragraph could also be expanded to emphasize potential research directions such as temporal modeling and domain adaptation, which are highly relevant to extending this work toward real-world applications. | Thank you for this valuable suggestion. The discussion section has been enhanced with deeper analytical insights linking performance trends to key design choices, including data augmentation strategies and transfer learning. Class-wise performance differences and representative misdetection examples are now discussed to improve interpretability, and the future work section has been expanded to highlight directions such as temporal modeling and domain adaptation for real-world deployment. |
| (6) Figures, Tables, and Formatting  There are some presentation issues that should be addressed. The formatting of Tables 1 and 2 is inconsistent in terms of font, spacing, and line thickness; they should be revised to conform with the journal’s style guide. Some tables are overly dense and could be simplified by merging or reorganizing columns. Higher-resolution detection visualizations would better demonstrate the model’s effectiveness. Terminology should be consistent throughout — use “YOLOv8-n” uniformly, avoiding variants such as “YOLOv8n” or “YOLOv8 nano.” | Thank you for pointing out these presentation details. All tables have been reformatted to comply with the journal’s style guidelines, with improved spacing, consistent fonts, and simplified layouts where appropriate. High-resolution detection visualizations have been updated, and terminology has been standardized throughout the manuscript using “YOLOv8-n” for consistency. |
| (7) Writing and Language Quality  The manuscript is generally well written in English, with clear logic and good readability. However, several sentences are overly long, and some word choices are repetitive. A light round of language polishing is recommended to improve conciseness and fluency. Final proofreading by a native speaker would further refine the manuscript and ensure consistency with international publication standards. | Thank you for this helpful suggestion. The manuscript has undergone a thorough language revision to improve conciseness, fluency, and sentence structure, with redundant expressions reduced throughout the text. Additional proofreading has been carried out to ensure clarity, consistency, and alignment with international publication standards. |

| **Reviewer #2 Comment** | **Author Response** |
| --- | --- |
| 1. In recent years, object detection algorithms have advanced rapidly. Why did the authors not consider more accurate and faster models such as YOLOv11, YOLOv12, or the latest YOLOv13? Please clarify the rationale for choosing YOLOv8-n. | Thank you for this insightful question. The revised manuscript clarifies that YOLOv8-n was deliberately selected due to its architectural maturity, deployment stability, and well-established performance on resource-constrained edge platforms. Its strong accuracy–latency trade-off and reproducibility make it particularly suitable for real-time autonomous driving applications, allowing the study to focus on task-specific optimization rather than rapidly evolving experimental architectures. |
| 2. It is recommended to include extensive experiments comparing the proposed method with other mainstream methods to fully validate its effectiveness. | Thank you for this constructive recommendation. The revised manuscript now includes comparative experiments with representative mainstream lightweight detectors to clearly demonstrate the effectiveness of the proposed framework. These results highlight the balance achieved between detection accuracy, inference speed, and computational efficiency under identical experimental settings. |
| 3. Please provide detailed information about the merged dataset, including object types, number of images, and other relevant details. | Thank you for this helpful request. The manuscript has been updated to include detailed information about the merged dataset, including the object categories, total number of images, class-wise distribution, and key preprocessing steps. These additions improve the transparency, reproducibility, and clarity of the experimental design. |
| 4．Figure 5 only shows the training loss curve and lacks the validation loss, making it impossible to assess whether the model is overfitting. Please add the validation loss curve. | Thank you for highlighting this important point. The revised manuscript now includes the validation loss curve alongside the training loss, enabling direct assessment of overfitting behavior. The close alignment between the two curves demonstrates stable convergence and strong generalization of the proposed model. |
| 5. The “Model Selection and Architecture” section provides only a brief description of the model structure and design; more detailed explanation is needed. | Thank you for this valuable suggestion. The “Model Selection and Architecture” section has been expanded to provide a clearer and more detailed explanation of the network structure, key architectural components, and design rationale. These revisions improve technical clarity and better illustrate how the model supports real-time rare obstacle detection. |
| 6. The current model lacks innovation. Please clearly highlight the core contributions and innovations in the manuscript. | Thank you for this important comment. The manuscript has been revised to explicitly highlight the core contributions, emphasizing the task-specific optimization of a lightweight detector, the curated rare obstacle dataset, and the comprehensive real-time evaluation under resource-constrained settings. These clarifications strengthen the presentation of the work’s innovation and practical relevance. |
| 7. Please check and adjust the manuscript formatting according to the journal template to ensure compliance. | Thank you for noting this issue. The manuscript formatting has been carefully revised to comply with the journal’s template, including adjustments to fonts, spacing, section headings, and references. These changes ensure full consistency with the journal’s submission guidelines. |
| 8. Please review the figure and table layouts according to the journal template to ensure they are clear, standardized, and properly formatted. | Thank you for this helpful recommendation. All figures and tables have been revised to align with the journal template, ensuring standardized layouts, consistent styling, and improved readability. High-resolution visuals and properly formatted tables are now used throughout the manuscript. |

We sincerely thank the Academic Editor and the reviewers for their thorough evaluation and constructive comments, which have greatly helped improve the quality and clarity of our manuscript. We have carefully addressed all comments and revised the manuscript accordingly. We believe that the revisions have significantly strengthened the presentation, methodological clarity, and experimental validation of the study. We hope that the revised manuscript now meets the requirements for publication in *PLOS ONE*, and we appreciate your time and consideration.
