# Supplementary material for: Real-time detection of rare roadside obstacles using YOLOv8-n in autonomous vehicles
Source: PLoS One. 2026 Jun 12;21(6):e0350732. doi: 10.1371/journal.pone.0350732 (PMC13262933; doi:10.1371/journal.pone.0350732)
Supplement: Attachment — Submitted filename: Rebuttal_Letter_PLOSOne_auresp_2.docx [file pone.0350732.s003.docx]

**Response to Reviewers**

Manuscript ID: PONE-D-25-53932R1

Title: *Real-Time Detection of Rare Roadside Obstacles using YOLOv8-n in Autonomous Vehicles.*

We sincerely thank the Academic Editor and the reviewers for their valuable time, constructive feedback, and positive assessment of our manuscript. We are pleased that the reviewers found the manuscript significantly improved and close to publishable quality. We have carefully addressed all comments and revised the manuscript accordingly.

All changes have been incorporated in the revised manuscript, and a tracked version has been provided. Below, we provide a detailed, point-by-point response to each comment.

**Responses to Reviewer #1**

We thank the reviewer for the positive evaluation and helpful suggestions.

**Comment:** *“A few figures may benefit from improved resolution for better presentation quality.”*
**Response:** Thank you for this suggestion. We have improved the resolution and overall quality of all figures to meet PLOS ONE’s publication standards.

**Comment:** *“A brief statement on limitations or future work could strengthen the discussion.”*
**Response:** We appreciate this recommendation. A new paragraph discussing the limitations of the proposed approach and potential future research directions has been added to the Discussion and Practical Implications section (5.7).

**Comment:** *“A light proofreading pass is recommended…”*

**Response:** The manuscript has undergone thorough proofreading to correct grammatical issues and ensure consistency in formatting and language throughout.

**Responses to Reviewer #2**

We thank the reviewer for the detailed and constructive feedback, which has helped us further improve the manuscript.

**Comment 1:** *Section numbering is missing.*

**Response:** We have revised the manuscript to include proper numbering for all sections and subsections in accordance with the journal’s formatting guidelines.

**Comment 2:** *Redundant tables (Table 3 and Table 4).*

**Response:** We thank the reviewer for this valuable suggestion. The redundant Table 4 has been retained and renumbered as Table 3, while the original Table 3 has been removed to avoid duplication and ensure consistency in the manuscript.

**Comment 3:** *Inconsistent training epochs (50 vs 200).*

**Response:** We appreciate this observation. The inconsistency has been corrected, and the training configuration is now consistently reported as 300 epochs throughout the manuscript, particularly in the Experimental Settings section.

**Comment 4:** *Inaccurate description of YOLOv8-n architecture.*

**Response:** We sincerely thank the reviewer for this important observation. We acknowledge that the previous version of the manuscript did not clearly distinguish between the original YOLOv8-n architecture and the modifications introduced in our proposed approach. In the revised manuscript, we have corrected the description of the YOLOv8-n model and clarified that the feature-weighted fusion formulation presented in Eq. (3) is part of the proposed enhancement introduced in this work, rather than a component of the vanilla YOLOv8-n architecture. The related text, equations, and architectural descriptions have been revised accordingly to avoid ambiguity and to more accurately reflect the contribution of the proposed method (section 3.2 updated)

**Comment 5:** *Lack of explicit comparison between proposed model and vanilla YOLOv8-n.*
**Response:** We thank the reviewer for this valuable suggestion. A detailed comparison between the baseline YOLOv8-n and the proposed YOLOv8-n model is already provided in Table 3 and further discussed in the Results and Discussion sections. However, to improve clarity and better highlight the contribution of the proposed method, we have revised the manuscript to explicitly emphasize the differences between the two models in both the text and table description. These revisions make the novelty and improvements of the proposed approach clearer to the reader (Section 5.7)

**Comment 6:** *Formatting issues (misaligned margins).*

**Response:** The formatting of the manuscript has been thoroughly revised to ensure proper alignment and compliance with the journal template.

We believe that these revisions have significantly improved the clarity, technical accuracy, and presentation of the manuscript. We sincerely hope that the revised version meets the journal’s requirements and is suitable for publication in PLOS ONE.

Thank you again for your time and consideration.

Kind regards,

**Afeera Bint-e-Tanveer**

(On behalf of all authors)
