# Supplementary material for: Real-time detection of rare roadside obstacles using YOLOv8-n in autonomous vehicles
Source: PLoS One. 2026 Jun 12;21(6):e0350732. doi: 10.1371/journal.pone.0350732 (PMC13262933; doi:10.1371/journal.pone.0350732)
Supplement: Peer review history

# Peer review: Decision Letter 0

**DOI:** 10.1371/journal.pone.0350732.r001

**Authors:** Ryan Wen Liu

18 Nov 2025

PONE-D-25-53932

Real-Time detection of rare roadside obstacles using YOLOv8-n in Autonomous Vehicles

PLOS ONE

Dear Dr. Tanveer,

Thank you for submitting your manuscript to PLOS ONE. After careful consideration, we feel that it has merit but does not fully meet PLOS ONE's publication criteria as it currently stands. Therefore, we invite you to submit a revised version of the manuscript that addresses the points raised during the review process.

Please submit your revised manuscript by January 3 2026 11:59 PM. If you will need more time than this to complete your revisions, please reply to this message or contact the journal office at plosone@plos.org. When you're ready to submit your revision, log on to https://www.editorialmanager.com/pone/ and select the 'Submissions Needing Revision' folder to locate your manuscript file.

Please include the following items when submitting your revised manuscript:

- A rebuttal letter that responds to each point raised by the academic editor and reviewer(s). You should upload this letter as a separate file labeled 'Response to Reviewers'.
- A marked-up copy of your manuscript that highlights changes made to the original version. You should upload this as a separate file labeled 'Revised Manuscript with Track Changes'.
- An unmarked version of your revised paper without tracked changes. You should upload this as a separate file labeled 'Manuscript'.

If you would like to make changes to your financial disclosure, please include your updated statement in your cover letter. Guidelines for resubmitting your figure files are available below the reviewer comments at the end of this letter.

If applicable, we recommend that you deposit your laboratory protocols in protocols.io to enhance the reproducibility of your results. Protocols.io assigns your protocol its own identifier (DOI) so that it can be cited independently in the future. For instructions see: https://journals.plos.org/plosone/s/submission-guidelines#loc-laboratory-protocols. Additionally, PLOS ONE offers an option for publishing peer-reviewed Lab Protocol articles, which describe protocols hosted on protocols.io. Read more information on sharing protocols at https://plos.org/protocols?utm_medium=editorial-email&utm_source=authorletters&utm_campaign=protocols.

We look forward to receiving your revised manuscript.

Kind regards,

Ryan Wen Liu

Academic Editor

PLOS ONE

Journal Requirements:

When submitting your revision, we need you to address these additional requirements.

1. Please ensure that your manuscript meets PLOS ONE's style requirements, including those for file naming. The PLOS ONE style templates can be found at

https://journals.plos.org/plosone/s/file?id=wjVg/PLOSOne_formatting_sample_main_body.pdf and

https://journals.plos.org/plosone/s/file?id=ba62/PLOSOne_formatting_sample_title_authors_affiliations.pdf

2. Please note that PLOS One has specific guidelines on code sharing for submissions in which author-generated code underpins the findings in the manuscript. In these cases, we expect all author-generated code to be made available without restrictions upon publication of the work. Please review our guidelines at https://journals.plos.org/plosone/s/materials-and-software-sharing#loc-sharing-code and ensure that your code is shared in a way that follows best practice and facilitates reproducibility and reuse.

3. If the reviewer comments include a recommendation to cite specific previously published works, please review and evaluate these publications to determine whether they are relevant and should be cited. There is no requirement to cite these works unless the editor has indicated otherwise.

Additional Editor Comments :

Prepare a major revision and resubmit:

1. Please provide a clearer clarification of the innovation of this work.

2. Please address the reviewers' requests for additional experiments.

[Note: HTML markup is below. Please do not edit.]

Reviewers' comments:

Reviewer's Responses to Questions

**Comments to the Author**

1. Is the manuscript technically sound, and do the data support the conclusions?

The manuscript must describe a technically sound piece of scientific research with data that supports the conclusions. Experiments must have been conducted rigorously, with appropriate controls, replication, and sample sizes. The conclusions must be drawn appropriately based on the data presented.

Reviewer #1: Yes

Reviewer #2: Partly

**********

2. Has the statistical analysis been performed appropriately and rigorously?

Reviewer #1: Yes

Reviewer #2: No

**********

3. Have the authors made all data underlying the findings in their manuscript fully available?

The PLOS Data policy requires authors to make all data underlying the findings described in their manuscript fully available without restriction, with rare exception (please refer to the Data Availability Statement in the manuscript PDF file). The data should be provided as part of the manuscript or its supporting information, or deposited to a public repository. For example, in addition to summary statistics, the data points behind means, medians and variance measures should be available. If there are restrictions on publicly sharing data—e.g. participant privacy or use of data from a third party—those must be specified.

Reviewer #1: Yes

Reviewer #2: No

**********

4. Is the manuscript presented in an intelligible fashion and written in standard English?

PLOS ONE does not copyedit accepted manuscripts, so the language in submitted articles must be clear, correct, and unambiguous. Any typographical or grammatical errors should be corrected at revision, so please note any specific errors here.

Reviewer #1: Yes

Reviewer #2: Yes

**********

5. Review Comments to the Author

Please use the space provided to explain your answers to the questions above. You may also include additional comments for the author, including concerns about dual publication, research ethics, or publication ethics. (Please upload your review as an attachment if it exceeds 20,000 characters)

Reviewer #1: Manuscript ID: PONE-D-25-53932

Title: Real-Time detection of rare roadside obstacles using YOLOv8-n in Autonomous Vehicles

Recommendation: Minor Revision

This manuscript proposes a lightweight real-time detection framework based on YOLOv8-n for identifying rare roadside obstacles in autonomous driving environments, including traffic cones, branches, barrels, debris, and rocks. The research topic is practically significant and addresses a safety-critical gap in autonomous perception, recognizing infrequent and atypical obstacles under computational constraints. The proposed framework balances detection accuracy and efficiency, and the overall paper is clearly structured, logically coherent, and methodologically sound. The experimental results are comprehensive and convincing. However,

(1) Novelty and Academic Contribution

In terms of technical originality, the manuscript primarily reflects engineering optimization and integration rather than algorithmic innovation. The authors are encouraged to clarify the originality of their contributions, for example, whether any modifications have been made to feature fusion, detection heads, loss functions, or network pruning strategies within the YOLOv8-n framework. If the approach mainly relies on fine-tuning and transfer learning, the paper could include a brief comparison table or quantitative discussion showing differences in model parameters, inference speed, and accuracy between the proposed framework and the vanilla YOLOv8-n. Additionally, the rationale for choosing YOLOv8-n instead of other lightweight versions such as YOLOv5s, YOLOv7-tiny, or MobileNet-SSD should be better articulated to strengthen methodological justification.

(2) Dataset and Experimental Design

The dataset construction and experimental setup demonstrate a solid engineering workflow. The authors combined several open-source datasets, applied consistent image normalization and resizing, and performed data augmentation (e.g., brightness variation, rotation, and geometric transformation). However, the dataset description is somewhat vague, only stating that the images were collected from Roboflow. It is strongly recommended that the authors specify the dataset names, number of images, class distribution, and data-cleaning procedures to enhance reproducibility. If specific augmentation techniques were used, they should be explicitly described. In cases where the full dataset cannot be released, including sample statistics or representative images in an appendix would significantly improve transparency and replicability.

(3) Methodology and Algorithm Details

The methodology section is clearly written and logically organized. The description of the CSPDarkNet backbone, PANet feature fusion, and detection heads is complete, and the mathematical formulations are concise and correct. Nevertheless, Algorithm 1 appears somewhat redundant. The initialization and fine-tuning phases could be merged, and the multi-step post-processing in the inference stage could be condensed into a unified detection loop. Simplifying the pseudocode would make it more professional and readable. Moreover, adding a flowchart that visualizes the entire process, from input images to final detection outputs, would improve the paper’s technical clarity. For the transfer learning process, more training details should be provided, such as whether backbone layers were frozen and whether differential learning rates were applied during fine-tuning.

(4) Experiments and Result Analysis

The experimental section is one of the highlights of this paper. The performance evaluation is comprehensive, including Precision, Recall, F1-score, and mAP@0.5, with clear visualizations of training curves and confusion matrices. The model achieved excellent results (Precision = 95.4%, Recall = 93.9%, F1 = 94.6%, mAP@0.5 = 98.1%) and demonstrated real-time inference capability at 68 FPS on an NVIDIA P100 GPU. These results strongly support the effectiveness of the proposed framework. To further strengthen the empirical analysis, the authors should include a lightweight baseline (such as YOLOv8s or YOLOv5s) for direct comparison, even if only briefly summarized. This will make the performance gain more explicit. If multiple training runs were conducted, the authors could report the mean and standard deviation; otherwise, note that the results are based on a single run. Additionally, a short ablation analysis—such as showing performance drops without data augmentation or transfer learning—would effectively demonstrate the contribution of each module.

(5) Discussion

The discussion section is complete but somewhat descriptive. It would benefit from deeper analysis and interpretive commentary. The authors should not only restate the results but also explain the underlying causes of performance differences. For instance, they could discuss which design choices (augmentation strategy, fine-tuning approach, feature integration) most influenced accuracy and why some object classes (e.g., debris vs. barrels) performed differently. Including one or two examples of misdetections or false negatives, along with brief visual explanations (e.g., due to occlusion or poor lighting), would substantially enhance the paper’s credibility. The future work paragraph could also be expanded to emphasize potential research directions such as temporal modeling and domain adaptation, which are highly relevant to extending this work toward real-world applications.

(6) Figures, Tables, and Formatting

There are some presentation issues that should be addressed. The formatting of Tables 1 and 2 is inconsistent in terms of font, spacing, and line thickness; they should be revised to conform with the journal’s style guide. Some tables are overly dense and could be simplified by merging or reorganizing columns. Higher-resolution detection visualizations would better demonstrate the model’s effectiveness. Terminology should be consistent throughout — use “YOLOv8-n” uniformly, avoiding variants such as “YOLOv8n” or “YOLOv8 nano.”

(7) Writing and Language Quality

The manuscript is generally well written in English, with clear logic and good readability. However, several sentences are overly long, and some word choices are repetitive. A light round of language polishing is recommended to improve conciseness and fluency. Final proofreading by a native speaker would further refine the manuscript and ensure consistency with international publication standards.

In conclusion, this paper presents a well-motivated, methodologically solid, and experimentally validated study addressing a meaningful real-world problem. Although its algorithmic innovation is moderate, the work has significant engineering and application value for autonomous driving perception systems and edge AI deployment. After moderate revision—mainly clarifying novelty, expanding dataset details, improving pseudocode and formatting, and enriching the discussion with causal analysis and failure examples—the paper will reach publishable quality.

Reviewer #2: The authors propose a lightweight real-time detection framework based on YOLOv8-n, which can accurately identify rare roadside obstacles on resource-constrained hardware. However, the manuscript requires major revisions. Specific comments are as follows:

1. In recent years, object detection algorithms have advanced rapidly. Why did the authors not consider more accurate and faster models such as YOLOv11, YOLOv12, or the latest YOLOv13? Please clarify the rationale for choosing YOLOv8-n.

2. It is recommended to include extensive experiments comparing the proposed method with other mainstream methods to fully validate its effectiveness.

3. Please provide detailed information about the merged dataset, including object types, number of images, and other relevant details.

4. Figure 5 only shows the training loss curve and lacks the validation loss, making it impossible to assess whether the model is overfitting. Please add the validation loss curve.

5. The “Model Selection and Architecture” section provides only a brief description of the model structure and design; more detailed explanation is needed.

6. The current model lacks innovation. Please clearly highlight the core contributions and innovations in the manuscript.

7. Please check and adjust the manuscript formatting according to the journal template to ensure compliance.

8. Please review the figure and table layouts according to the journal template to ensure they are clear, standardized, and properly formatted.

**********

6. PLOS authors have the option to publish the peer review history of their article (what does this mean?). If published, this will include your full peer review and any attached files.

If you choose “no”, your identity will remain anonymous but your review may still be made public.

**Do you want your identity to be public for this peer review?**   For information about this choice, including consent withdrawal, please see our Privacy Policy.

Reviewer #1: No

Reviewer #2: No

**********

[NOTE: If reviewer comments were submitted as an attachment file, they will be attached to this email and accessible via the submission site. Please log into your account, locate the manuscript record, and check for the action link "View Attachments". If this link does not appear, there are no attachment files.]

To ensure your figures meet our technical requirements, please review our figure guidelines: https://journals.plos.org/plosone/s/figures

You may also use PLOS’s free figure tool, NAAS, to help you prepare publication quality figures: https://journals.plos.org/plosone/s/figures#loc-tools-for-figure-preparation.

NAAS will assess whether your figures meet our technical requirements by comparing each figure against our figure specifications.

# Author comment: Author response to Decision Letter 1

**DOI:** 10.1371/journal.pone.0350732.r002

9 Jan 2026

We thank the Academic Editor and the reviewers for their constructive and insightful comments, which have helped improve the quality and clarity of our manuscript.

We have carefully addressed all reviewer and editor comments and submitted: (i) a detailed point-by-point rebuttal letter, (ii) a revised manuscript with tracked changes highlighting all modifications, and (iii) a clean revised manuscript. The revisions include clearer articulation of the contributions and innovation, expanded dataset and methodological descriptions, additional experimental analyses and comparisons, inclusion of validation loss curves, and improvements to figures, tables, and overall formatting in accordance with the PLOS ONE guidelines.

We hope that the revised manuscript now meets the journal’s requirements and look forward to your kind consideration.

Attachment Submitted filename: Rebuttal letter Plosone.docx

# Peer review: Decision Letter 1

**DOI:** 10.1371/journal.pone.0350732.r003

**Authors:** Ryan Wen Liu

29 Jan 2026

PONE-D-25-53932R1

Real-Time detection of rare roadside obstacles using YOLOv8-n in Autonomous Vehicles

PLOS One

Dear Dr. Tanveer,

Thank you for submitting your manuscript to PLOS ONE. After careful consideration, we feel that it has merit but does not fully meet PLOS ONE’s publication criteria as it currently stands. Therefore, we invite you to submit a revised version of the manuscript that addresses the points raised during the review process.

Please submit your revised manuscript by Mar 15 2026 11:59PM. If you will need more time than this to complete your revisions, please reply to this message or contact the journal office at plosone@plos.org. When you're ready to submit your revision, log on to https://www.editorialmanager.com/pone/ and select the 'Submissions Needing Revision' folder to locate your manuscript file.

Please include the following items when submitting your revised manuscript:

- A letter that responds to each point raised by the academic editor and reviewer(s). You should upload this letter as a separate file labeled 'Response to Reviewers'.
- A marked-up copy of your manuscript that highlights changes made to the original version. You should upload this as a separate file labeled 'Revised Manuscript with Track Changes'.
- An unmarked version of your revised paper without tracked changes. You should upload this as a separate file labeled 'Manuscript'.

If you would like to make changes to your financial disclosure, please include your updated statement in your cover letter. Guidelines for resubmitting your figure files are available below the reviewer comments at the end of this letter.

If applicable, we recommend that you deposit your laboratory protocols in protocols.io to enhance the reproducibility of your results. Protocols.io assigns your protocol its own identifier (DOI) so that it can be cited independently in the future. For instructions see: https://journals.plos.org/plosone/s/submission-guidelines#loc-laboratory-protocols. Additionally, PLOS ONE offers an option for publishing peer-reviewed Lab Protocol articles, which describe protocols hosted on protocols.io. Read more information on sharing protocols at https://plos.org/protocols?utm_medium=editorial-email&utm_source=authorletters&utm_campaign=protocols.

We look forward to receiving your revised manuscript.

Kind regards,

Ryan Wen Liu

Academic Editor

PLOS One

Journal Requirements:

1. If the reviewer comments include a recommendation to cite specific previously published works, please review and evaluate these publications to determine whether they are relevant and should be cited. There is no requirement to cite these works unless the editor has indicated otherwise.

2. Please review your reference list to ensure that it is complete and correct. If you have cited papers that have been retracted, please include the rationale for doing so in the manuscript text, or remove these references and replace them with relevant current references. Any changes to the reference list should be mentioned in the rebuttal letter that accompanies your revised manuscript. If you need to cite a retracted article, indicate the article’s retracted status in the References list and also include a citation and full reference for the retraction notice.

Additional Editor Comments:

Prepare a minor revision and resubmit:

Please ensure that the reviewers' comments are duly addressed.

[Note: HTML markup is below. Please do not edit.]

Reviewers' comments:

Reviewer's Responses to Questions

**Comments to the Author**

1. If the authors have adequately addressed your comments raised in a previous round of review and you feel that this manuscript is now acceptable for publication, you may indicate that here to bypass the “Comments to the Author” section, enter your conflict of interest statement in the “Confidential to Editor” section, and submit your "Accept" recommendation.

Reviewer #1: All comments have been addressed

Reviewer #2: (No Response)

**********

2. Is the manuscript technically sound, and do the data support the conclusions?

The manuscript must describe a technically sound piece of scientific research with data that supports the conclusions. Experiments must have been conducted rigorously, with appropriate controls, replication, and sample sizes. The conclusions must be drawn appropriately based on the data presented.

Reviewer #1: Yes

Reviewer #2: (No Response)

**********

3. Has the statistical analysis been performed appropriately and rigorously?

Reviewer #1: Yes

Reviewer #2: (No Response)

**********

4. Have the authors made all data underlying the findings in their manuscript fully available?

The PLOS Data policy requires authors to make all data underlying the findings described in their manuscript fully available without restriction, with rare exception (please refer to the Data Availability Statement in the manuscript PDF file). The data should be provided as part of the manuscript or its supporting information, or deposited to a public repository. For example, in addition to summary statistics, the data points behind means, medians and variance measures should be available. If there are restrictions on publicly sharing data—e.g. participant privacy or use of data from a third party—those must be specified.

Reviewer #1: Yes

Reviewer #2: (No Response)

**********

5. Is the manuscript presented in an intelligible fashion and written in standard English?

PLOS ONE does not copyedit accepted manuscripts, so the language in submitted articles must be clear, correct, and unambiguous. Any typographical or grammatical errors should be corrected at revision, so please note any specific errors here.

Reviewer #1: Yes

Reviewer #2: (No Response)

**********

6. Review Comments to the Author

Please use the space provided to explain your answers to the questions above. You may also include additional comments for the author, including concerns about dual publication, research ethics, or publication ethics. (Please upload your review as an attachment if it exceeds 20,000 characters)

Reviewer #1: The manuscript is significantly improved, most issues from the first-round review have been satisfactorily addressed. The manuscript is now close to publishable quality.

A few figures may benefit from improved resolution for better presentation quality.

A brief statement on limitations or future work could strengthen the discussion.

A light proofreading pass is recommended to ensure consistent formatting and grammar throughout.

Reviewer #2: The authors have adequately responded to most of the comments raised in the previous round of review, and the manuscript has been substantially improved in terms of structural completeness, clarity of methodological description, and experimental validation. The revised version shows better overall readability and technical rigor. However, several issues still require further revision and clarification:

1. Section numbering: Currently, the manuscript does not provide numbering for sections, and subsections are also not numbered, which may cause confusion when reading and referencing. The authors are advised to add proper numbering for all sections and subsections in accordance with the journal’s formatting requirements.

2. Redundant tables: Table 4 contains the same information as Table 3, resulting in redundancy. The authors are recommended to remove the redundant table or merge Tables 3 and 4 to avoid repeating the same data.

3. Inconsistent training epochs: Different parts of the manuscript report training for 50 epochs and 200 epochs, which is inconsistent. The authors should unify the number of training epochs and related hyperparameters and provide a clear and consistent description in the “Experimental Settings” section.

4. Inaccurate description of the YOLOv8-n architecture: The description of YOLOv8-n in the manuscript is not fully accurate. For example, the feature-weighted fusion formulation in Eq. (3) does not correspond to the original YOLOv8-n architecture. If this fusion strategy is part of the authors’ proposed method, it should be clearly stated as a modification introduced in this work rather than as a component of the vanilla YOLOv8-n model, and the related descriptions should be revised accordingly.

5. Lack of explicit comparison between models: The authors are encouraged to clearly describe the specific differences between the proposed YOLOv8-n and the vanilla YOLOv8-n, so that readers can better understand the novelty and contributions of the proposed approach.

6. Formatting issues: The right margin of the manuscript is noticeably misaligned, which affects the overall layout quality and readability. The authors should revise the formatting of the entire manuscript to fully comply with the journal template.

**********

7. PLOS authors have the option to publish the peer review history of their article (what does this mean?). If published, this will include your full peer review and any attached files.

If you choose “no”, your identity will remain anonymous but your review may still be made public.

**Do you want your identity to be public for this peer review?**   For information about this choice, including consent withdrawal, please see our Privacy Policy.

Reviewer #1: No

Reviewer #2: No

**********

[NOTE: If reviewer comments were submitted as an attachment file, they will be attached to this email and accessible via the submission site. Please log into your account, locate the manuscript record, and check for the action link "View Attachments". If this link does not appear, there are no attachment files.]

To ensure your figures meet our technical requirements, please review our figure guidelines: https://journals.plos.org/plosone/s/figures

You may also use PLOS’s free figure tool, NAAS, to help you prepare publication quality figures: https://journals.plos.org/plosone/s/figures#loc-tools-for-figure-preparation.

NAAS will assess whether your figures meet our technical requirements by comparing each figure against our figure specifications.

# Author comment: Author response to Decision Letter 2

**DOI:** 10.1371/journal.pone.0350732.r004

12 May 2026

We sincerely thank the Editor and the reviewers for their valuable and constructive comments. We have carefully revised the manuscript in response to all suggestions. We provide a point-by-point response to each comment in the rebuttal letter. We are grateful for the opportunity to submit our work to PLOS ONE and appreciate the time and effort invested in reviewing our manuscript. We look forward to any further feedback and hope that the revised manuscript meets the journal’s standards for publication.

Attachment Submitted filename: Rebuttal_Letter_PLOSOne_auresp_2.docx

# Peer review: Decision Letter 2

**DOI:** 10.1371/journal.pone.0350732.r005

**Authors:** Ryan Wen Liu

19 May 2026

Real-Time detection of rare roadside obstacles using YOLOv8-n in Autonomous Vehicles

PONE-D-25-53932R2

Dear Dr. Tanveer,

We’re pleased to inform you that your manuscript has been judged scientifically suitable for publication and will be formally accepted for publication once it meets all outstanding technical requirements.

Within one week, you’ll receive an e-mail detailing the required amendments. When these have been addressed, you’ll receive a formal acceptance letter and your manuscript will be scheduled for publication.

An invoice will be generated when your article is formally accepted. Please note, if your institution has a publishing partnership with PLOS and your article meets the relevant criteria, all or part of your publication costs will be covered. Please make sure your user information is up-to-date by logging into Editorial Manager at Editorial Manager® and clicking the ‘Update My Information' link at the top of the page. For questions related to billing, please contact billing support.

If your institution or institutions have a press office, please notify them about your upcoming paper to help maximize its impact. If they’ll be preparing press materials, please inform our press team as soon as possible -- no later than 48 hours after receiving the formal acceptance. Your manuscript will remain under strict press embargo until 2 pm Eastern Time on the date of publication. For more information, please contact onepress@plos.org.

Kind regards,

Ryan Wen Liu

Academic Editor

PLOS One

Additional Editor Comments (optional):

It is necessary to carefully check and revise the potential errors before the final submission.

Reviewers' comments:

Reviewer's Responses to Questions

**Comments to the Author**

1. If the authors have adequately addressed your comments raised in a previous round of review and you feel that this manuscript is now acceptable for publication, you may indicate that here to bypass the “Comments to the Author” section, enter your conflict of interest statement in the “Confidential to Editor” section, and submit your "Accept" recommendation.

Reviewer #2: (No Response)

**********

2. Is the manuscript technically sound, and do the data support the conclusions?

The manuscript must describe a technically sound piece of scientific research with data that supports the conclusions. Experiments must have been conducted rigorously, with appropriate controls, replication, and sample sizes. The conclusions must be drawn appropriately based on the data presented.

Reviewer #2: (No Response)

**********

3. Has the statistical analysis been performed appropriately and rigorously?

Reviewer #2: (No Response)

**********

4. Have the authors made all data underlying the findings in their manuscript fully available?

The PLOS Data policy requires authors to make all data underlying the findings described in their manuscript fully available without restriction, with rare exception (please refer to the Data Availability Statement in the manuscript PDF file). The data should be provided as part of the manuscript or its supporting information, or deposited to a public repository. For example, in addition to summary statistics, the data points behind means, medians and variance measures should be available. If there are restrictions on publicly sharing data—e.g. participant privacy or use of data from a third party—those must be specified.

Reviewer #2: (No Response)

**********

5. Is the manuscript presented in an intelligible fashion and written in standard English?

PLOS ONE does not copyedit accepted manuscripts, so the language in submitted articles must be clear, correct, and unambiguous. Any typographical or grammatical errors should be corrected at revision, so please note any specific errors here.

Reviewer #2: (No Response)

**********

6. Review Comments to the Author

Please use the space provided to explain your answers to the questions above. You may also include additional comments for the author, including concerns about dual publication, research ethics, or publication ethics. (Please upload your review as an attachment if it exceeds 20,000 characters)

Reviewer #2: The authors have addressed all the issues I raised satisfactorily. I agree to accept the paper and recommend it for publication.

**********

7. PLOS authors have the option to publish the peer review history of their article (what does this mean?). If published, this will include your full peer review and any attached files.

If you choose “no”, your identity will remain anonymous but your review may still be made public.

**Do you want your identity to be public for this peer review?**   For information about this choice, including consent withdrawal, please see our Privacy Policy.

Reviewer #2: No

**********

# Editor report: Acceptance letter

**DOI:** 10.1371/journal.pone.0350732.r006

**Authors:** Ryan Wen Liu

PONE-D-25-53932R2

PLOS One

Dear Dr. Tanveer,

I'm pleased to inform you that your manuscript has been deemed suitable for publication in PLOS One. Congratulations! Your manuscript is now being handed over to our production team.

At this stage, our production department will prepare your paper for publication. This includes ensuring the following:

* All references, tables, and figures are properly cited

* All relevant supporting information is included in the manuscript submission,

* There are no issues that prevent the paper from being properly typeset

You will receive further instructions from the production team, including instructions on how to review your proof when it is ready. Please keep in mind that we are working through a large volume of accepted articles, so please give us a few days to review your paper and let you know the next and final steps.

Lastly, if your institution or institutions have a press office, please let them know about your upcoming paper now to help maximize its impact. If they'll be preparing press materials, please inform our press team within the next 48 hours. Your manuscript will remain under strict press embargo until 2 pm Eastern Time on the date of publication. For more information, please contact onepress@plos.org.

You will receive an invoice from PLOS for your publication fee after your manuscript has reached the completed accept phase. If you receive an email requesting payment before acceptance or for any other service, this may be a phishing scheme. Learn how to identify phishing emails and protect your accounts at https://explore.plos.org/phishing.

If we can help with anything else, please email us at customercare@plos.org.

Thank you for submitting your work to PLOS ONE and supporting open access.

Kind regards,

PLOS ONE Editorial Office Staff

on behalf of

Professor Ryan Wen Liu

Academic Editor

PLOS One
